# Tunica vaginalis graft use for repair of testicular rupture after blunt trauma: A report of two cases and literature review

**DOI:** 10.1016/j.ijscr.2025.111754

**Published:** 2025-08-05

**Authors:** Xiangxiang Zhang, Fanfan Li, Mao Zhang, Hengping Li

**Affiliations:** a Department of Urology, Gansu Provincial Hospital, Lanzhou, Gansu, China; b Gansu University of Chinese Medicine, Lanzhou, China

**Keywords:** Testicular rupture, Tunica albuginea defects, Tunica vaginalis, Repair, Case report

## Abstract

**Background:**

Testicular rupture caused by blunt scrotal trauma can be repaired routinely, but for severe testicular rupture often accompanied by a large area of tunica albuginea defect, it is impossible to suture the tunica albuginea without tension unless excess normal testicular tissue is removed. This case report highlights the surgical challenge of using a pedicled tunica vaginalis in the case of severe tunica albuginea defect in testicular rupture.

**Case presentation:**

Two underage boys both experienced severe pain in the scrotum due to trauma, and ultrasound examination showed testicular rupture. Emergency surgical exploration was performed, and severe testicular rupture was observed during the operation, with a large area of tunica albuginea defect that could not be repaired routinely. After trimming the necrotic testicular tissue, a pedicled testicular sheath was sutured to the residual tunica albuginea. After one year of postoperative follow-up, the recovery was good and hormone levels were normal.

**Discussion:**

Surgical repair of testicular rupture presents certain challenges, especially for severe tunica albuginea defects. The use of a pedicled testicular tunica vaginalis can more easily solve this problem, but the location and size of the tunica vaginalis need to be designed during surgery, especially to ensure blood supply to the tunica vaginalis.

**Conclusion:**

For severe testicular rupture, the use of pedicle testicular sheath repair is a recommended option, but it requires certain surgical techniques.

## Highlights

• Severe testicular rupture, usually accompanied by extensive tunica albuginea defects, requires the removal of excess normal testicular tissue to ensure tension-free suturing.
• For testicular rupture with obvious tunica albuginea defects, using the tunica vaginalis as a repair substitute can minimize the loss of live testicular tissue and reduce the risk of fertility and hormonal dysfunction.
• During the operation, the location for collecting the tunica vaginalis should be designed based on the missing tunica albuginea, and the blood supply to the tunica vaginalis must be ensured to avoid necrosis.

## 1 Introduction

Scrotal trauma in young males represents less than 1 % of all trauma-related injuries, 1.5 % of which complicated with testicular rupture [1,2]. The standard treatment of testicular rupture remains early exploration and debridement to attempt testicular salvage [3]. The surgical technique involves evacuation of any hematoma, debridement of the extruded seminiferous tubules, and primary closure of the tunica albuginea [4]. However, for testicular rupture with tunica albuginea defect, it is difficult to close the tunica albuginea in the early stage, and using a pedicled tunica vaginalis may be a better choice. In this paper, we report two cases of testicular rupture using the tunica vaginalis to repair tunica albuginea defects. This case serves as a reminder for urologists to reflect on such diagnostic challenges to avoid similar errors in the future. This case report has been reported according to the revised SCARE guidelines, 2025 [5].

## 2 Cases presentation

### 2.1 Case 1

**Fig. 1:**
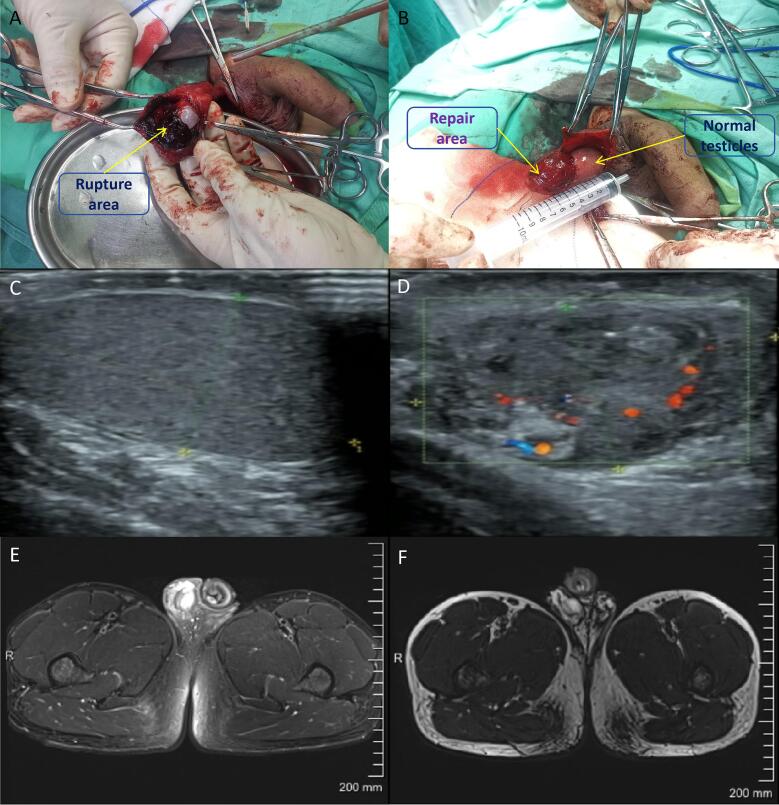
(A) Intraoperative tunica albuginea rupture was observed, with a rupture of approximately 1 cm. (B) Repair of tunica albuginea defect with tunica vaginalis, approximately 3 cm in length. (C) Six months after surgery, ultrasound examination revealed testicular morphology. (D) Six months after surgery, ultrasound showed good testicular blood supply. (E) Three months after surgery, MRI showed good testicular recovery. (F) Six months after surgery, MRI showed a reduction in testicular volume.

A 13-year-old boy was hit while playing with his playmate 4 days ago, causing swelling, pain, and discomfort in his right scrotum. Due to the unsatisfactory conservative treatment effect of self-oral anti-inflammatory drugs, the patient visited our hospital. Ultrasound examination showed right testicular and epididymal contusion and fissure. After admission, emergency surgery was performed. During the operation, two tunica albuginea ruptures and defects were observed in the right testicle, accompanied by a small amount of testicular necrotic tissue. Most of the testicular tissue was normal. After cleaning the necrotic testicular tissue, tunica vaginalis around the rupture site were selected to repair the tunica albuginea defect. The patient's scrotum showed no signs of infection or necrosis after surgery. B-ultrasound, MR, and hormone levels were rechecked at 6 and 12 months after surgery, respectively. The re-examination results all indicated that the volume of the right testicle was about 35 * 23 mm, with uneven echoes and richer blood flow signals compared to the contralateral side (Fig. 1). The testosterone level was about 17.62 nmol/L.

### 2.2 Case 2

**Fig. 2:**
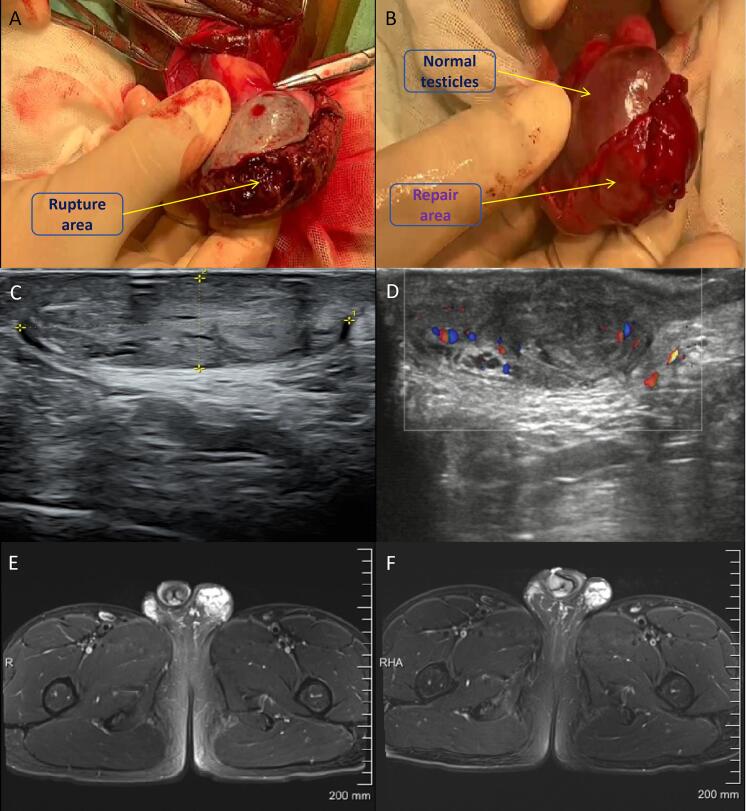
(A) During the operation, a large area of tunica albuginea defect and testicular parenchyma protrusion were observed in the testicles. (B) Repair the tunica albuginea defect with tunica vaginalis, with a length of approximately 4 cm. (C) Six months after surgery, B-ultrasound showed that the testicular morphology was still acceptable. (D) Six months after surgery, B-ultrasound showed good blood supply to the testicular repair site. (E) Three months after surgery, MRI showed good testicular recovery. (F) Six months after surgery, MRI showed a reduction in testicular volume.

A 17-year-old boy was hit while playing basketball half a day ago, causing pain and discomfort in his left scrotum. He sought medical attention at a local hospital and underwent pelvic CT, which revealed left testicular trauma. He was then transferred to our hospital for emergency surgery. During the operation, it was found that the left testicular tunica albuginea was severely ruptured, with nearly one-third of the testicular parenchyma extruded. After cleaning the blood swelling and trimming the necrotic testicular tissue, a pedicled tunica vaginalis was cut around the rupture site and sutured to the remaining end of the white membrane with 4–0 absorbable sutures to maintain a certain tension as much as possible. There was no significant necrosis or infection in the left scrotum of the postoperative patient. Follow up examinations at 6 and 12 months after surgery showed that the left testicle was approximately 37 * 20 mm, with uneven testicular echoes and richer blood flow signals on the left side compared to the contralateral side (Fig. 2). The testosterone level was about 16.14 nmol/L.

Both cases mentioned above were in a supine position and underwent general anesthesia. The surgeries were performed by experienced chief physicians. These two patients have severe testicular rupture and a large area of tunica albuginea defect, which cannot be primary closured. If the testicular tissue is removed and sutured directly, it may take away the viable testicular tissue and cause compartment syndrome, leading to testicular atrophy. After sufficient communication and agreement from the parents of the patient, we used the technique of pedicled testicular sheath to complete the surgery. During the operation, the tunica vaginalis was opened on the opposite side of the ruptured testicle to reduce the tension of the tunica vaginalis graft and ensure the width of the root of the graft, thereby better protecting the blood supply of the tunica vaginalis. There were no surgical complications observed during or after the operation in the pediatric patients.

## 3 Discussion

Rupture of the tunica albuginea exposes the seminiferous tubules which puts at stake prognosis for future fertility [6]. Severe testicular injury could affect the fertility and contribute to hypogonadism, which affects social confidence [7]. Testicular rupture with a defect in the tunica albuginea is considered difficult to place the protruding testicular tissue inside the tunica albuginea, even if the hematoma is cleared. Sacrificing viable seminiferous tubules may make primary closure possible, however, this primary closure can lead to interruption of blood flow and testicular necrosis [8]. Therefore, choosing to use testicular tunica vaginalis as an alternative tissue for repair and reconstruction is worth exploring and experimenting with.

As early as 1992, DEEPAK et al. first reported the use of the tunica vaginalis to solve the problem of tunica albuginea defects caused by testicular rupture [9]. And this technique was later reported in multiple studies, with a total of 11 patients using this method to treat testicular rupture, including some patients with penetrating testicular injury [4,8,[10], [11], [12], [13], [14]]. In addition, studies have shown that free grafts can be used to cover tunica albuginea defects. The success of free grafts depends on the blood flow of the graft bed, and recanalization is essential [8]. Polytetrafluoroethylene (PTFE) has been used for testicular capsule reconstruction after testicular trauma, but due to the high rate of graft infection, its effectiveness is poor, and in severe cases, the testicle needs to be removed [15]. The technology provided in this study does not require any special equipment or additional costs, avoids debridement of viable testicular tissue, and has an extremely low incidence of complications.

The surgery of using testicular tunica vaginalis to replace tunica albuginea defects requires certain skills and experience. Firstly, when preparing to cut the tunica vaginalis, we need to anticipate the approximate location of the testicular rupture as much as possible, and open the tunica vaginalis on the opposite side of the rupture. This can reserve more pedunculated tunica vaginalis to replace the potentially missing tunica albuginea, and suture the tunica vaginalis with the residual end of the ruptured tunica albuginea; In addition, if choosing to use the tunica vaginalis for repair, it is necessary to ensure the blood supply of the tunica vaginalis, avoid tunica vaginalis necrosis, cause hydrocele in the sheath cavity, wound leakage or rupture, and more worrying, it may lead to the formation of anti-sperm antibody, which in turn may pose a risk of infertility. Finally, when using tunica vaginalis repair, it is necessary to design the tunica vaginalis to be used based on the area of tunica albuginea defects after cleaning the necrotic testicular tissue, and to ensure that the tunica vaginalis flap has a certain tension after suturing, so as to limit the ruptured testicular tissue and promote healing.

## 4 Conclusion

For testicular rupture with obvious tunica albuginea defects, using the tunica vaginalis as a repair substitute can minimize the loss of live testicular tissue and reduce the risk of fertility and hormonal dysfunction. Preserving endocrine function and testicular volume is an important outcome of pediatric urology, and this technique can help us better achieve this goal.

## Consent

Written informed consent was obtained from the parents of minors for publication of this case report and accompanying images. A copy of the written consent is available for review by the Editor-in-Chief of this journal on request. The privacy of patient is protected in this article.

## Ethical approval

The case report was exempt from ethical approval by the Ethics Committee of Gansu Provincial Hospital, because we have obtained informed consent from the patient, which is not the design of the clinical trial. We have obtained informed consent from the patient and told the patient not at any risk to him. On the basis of full patient understanding and consent, informed consent was signed and consent was provided with the medical information to be used for scientific research and publications.

## Funding

This study was funded by the Research Fund Project of Gansu Provincial People's Hospital (Grant Nos. 23GSSYA-8), 10.13039/501100004775Natural Science Foundation of Gansu Province (Grant Nos. 22JR11RA271) and (Grant Nos. 25JRRA290), Lanzhou Science and Technology Plan Project (Grant Nos. 2024-9-12).

## Author contribution

Xiang-Xiang Zhang and Fan-Fan Li contributed equally to this work as co-first authors.

Constructing hypothesis for the manuscript: Heng-Ping Li and Xiang-Xiang Zhang.

Logical interpretation and presentation of the results: Mao Zhang.

Organizing and supervising the course of the article: Fan-Fan Li.

Construction of the whole or body of the manuscript and taking re sponsibility: Xiang-Xiang Zhang.

All the authors read and agreed with the published version of the manuscript.

## Guarantor

Xiangxiang Zhang.

## Research registration number

1. Name of the registry: Chinese Clinical Trial Registry chictr.org.cn

2. Unique identifying number or registration ID: ChiCTR2500108638

3. Hyperlink to your specific registration (must be publicly accessible and will be checked): https://www.chictr.org.cn/bin/home (用户名:张向向1993 密码:Zhangxx17@)

## Conflict of interest statement

There is no conflict of interest.

## References

[1] Deurdulian C., Mittelstaedt C.A., Chong W.K., Fielding J.R. (2007). US of acute scrotal trauma: optimal technique, imaging findings, and management. Radiographics.

[2] Bhatt S., Dogra V.S. (2008). Role of US in testicular and scrotal trauma. Radiographics.

[3] Wang Z., Yang J.R., Huang Y.M., Wang L., Liu L.F., Wei Y.B. (2016). Diagnosis and management of testicular rupture after blunt scrotal trauma: a literature review. Int. Urol. Nephrol..

[4] Jian P.Y., Nelson E.D., Roth D.R. (2012). Use of a vascularized tunica vaginalis flap for repair of testicular rupture in a pediatric patient. Urology.

[5] Revised Surgical CAse REport (SCARE) Guideline: An Update for the Age of Artificial Intelligence.

[6] Case R.I.E.M. (2019). Retracted: Marathon runner with acute hyponatremia: a neurological disorder. Case Rep Emerg Med..

[7] Addas F., Yan S., Hadjipavlou M., Gonsalves M., Sabbagh S. (2018). Testicular Rupture or Testicular Fracture? A Case Report and Literature Review. Case Rep. Urol..

[8] Yokokawa S., Tabei T., Kobayashi K. (2021). Testicular rupture successfully treated with a tunica vaginalis flap. IJU Case Rep..

[9] Kapoor D., Leech J., Yap W. (1992). Use of tunica vaginalis patch graft for repair of traumatic testicular rupture. Urology.

[10] Molokwu C.N., Doull R.I., Townell N.H. (2010). A novel technique for repair of testicular rupture after blunt trauma. Urology.

[11] Damle R.N., Lalikos J.F., Aidlen J.T., Ellsworth P. (2017). Tunica vaginalis pedicle flap for repair of ruptured testis: a single-center experience with four patients. J. Pediatr. Urol..

[12] Ahmed F., Naji M., Al-Hitari A., Amer Q., Al-Sagheer M., Chowdhury U. (2020). Use of tunica vaginalis graft for repair of traumatic bilateral testicular rupture after gunshot: a case report. Pan Afr. Med. J..

[13] Sadjo S.A., Destinval C., Lemelle J.L., Berte N. (2021). Testicular rupture after blunt scrotal trauma in children: a case report and literature review. Trauma Case Rep..

[14] Masih S., Damodaran S., DaJusta D.G. (2023). Tunica vaginalis graft use for repair of traumatic testicular rupture: a Case report. Urology.

[15] Ferguson G.G., Brandes S.B. (2007). Gunshot wound injury of the testis: the use of tunica vaginalis and polytetrafluoroethylene grafts for reconstruction. J. Urol..

